# Biological roles of RNA m7G modification and its implications in cancer

**DOI:** 10.1186/s13062-023-00414-5

**Published:** 2023-09-14

**Authors:** Xin Zhang, Wen-Yan Zhu, Shu-Yi Shen, Jia-Hao Shen, Xiao-Dong Chen

**Affiliations:** 1 Department of Dermatology, Affiliated Hospital of Nantong University, Medical School of Nantong University, Nantong, China; 2 Department of Dermatology, the First Affiliated Hospital of Fujian Medical University, Fuzhou, China

**Keywords:** N7-methylguanosine, Immune microenvironment, RNA methylation modification, Carcinoma

## Abstract

M7G modification, known as one of the common post-transcriptional modifications of RNA, is present in many different types of RNAs. With the accurate identification of m7G modifications within RNAs, their functional roles in the regulation of gene expression and different physiological functions have been revealed. In addition, there is growing evidence that m7G modifications are crucial in the emergence of cancer. Here, we review the most recent findings regarding the detection techniques, distribution, biological functions and Regulators of m7G. We also summarize the connections between m7G modifications and cancer development, drug resistance, and tumor microenvironment as well as we discuss the research’s future directions and trends.

## Introduction

**Fig. 1:**
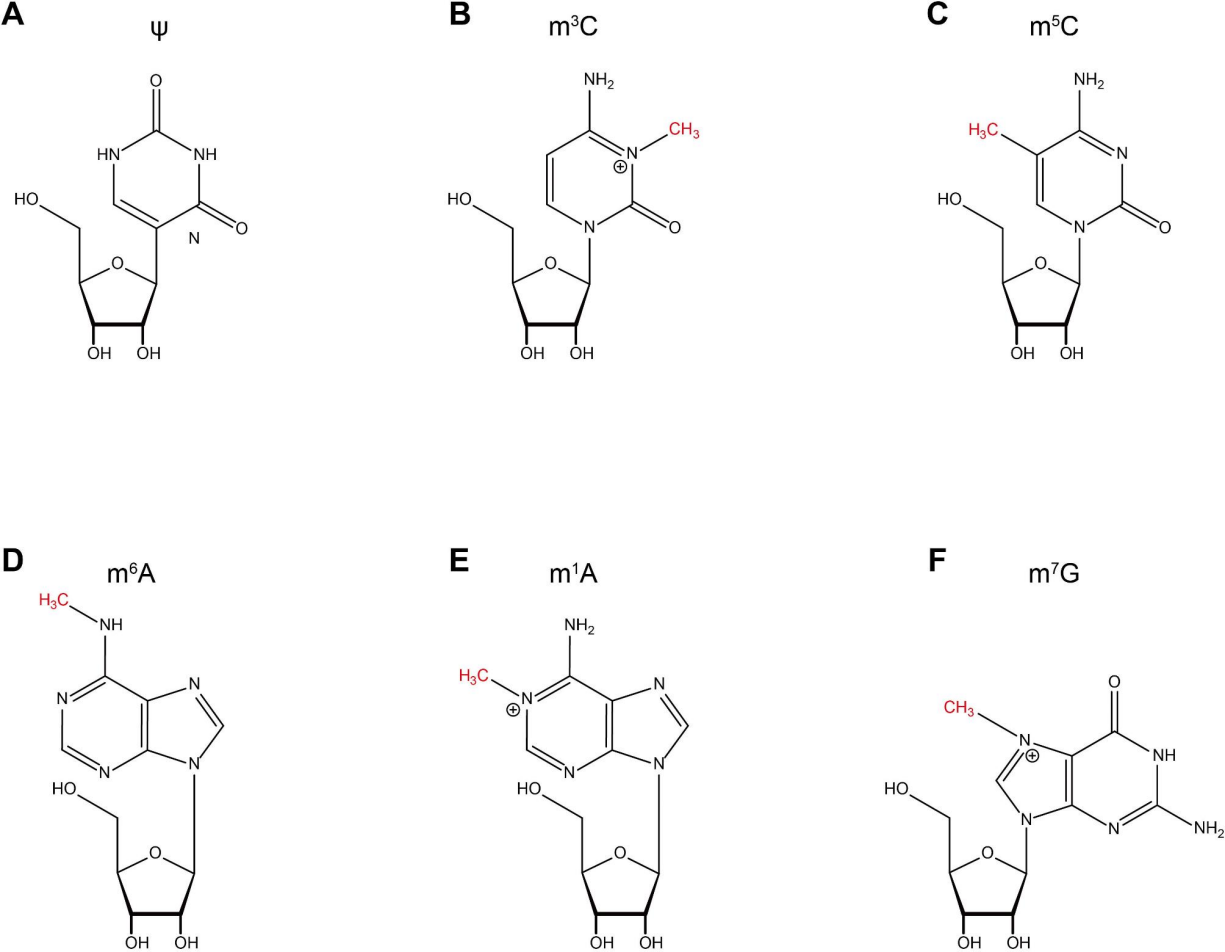
Molecular formula of common RNA modifications. (**A**) Ψ. (**B**) m3C. (**C**) m5C. (**D**) m6A. (**E**) m1A. (**F**) m7G

Epigenetics refers to heritable changes in gene expression without changing the nucleotide sequence of the gene, including DNA methylation, histone modifications, and RNA modifications. In recent years, RNA modifications have become a new research hotspot, and more than 170 RNA modifications have been identified [1], including N6-methyladenosine (M6A), N7-methylguanosine (M7G), 5-methylcytosine (M5C), N1-methyladenosine (M1A), N3-methylcytosine (M3C) and pseudouridine (ψ) [2] (Fig. 1), which play important roles in biological processes such as RNA metabolism and post-transcriptional regulation. With the rapid development of sequencing technology, m7G has become a new research hotspot of RNA modification.

Positively charged m7G modifications are frequently observed in eukaryotes, prokaryotes and archaea [3, 4]. M7G modifications are commonly found in mRNA, tRNA, rRNA, miRNA, and the 5′ cap end of eukaryotic mRNA [5, 6], which affects almost the whole process of RNA metabolism, including pre-mRNA splicing, stabilization of mRNA structure, transcription, translation, and nuclear export [4, 7–10]. In 2019, Zhang et al. comfirmed the existence of m7G inside mammalian mRNA for the first time and pioneered the invention of a new type of epigenetic sequencing method (m7G-seq) to reveal the distribution sites of m7G modifications [11], which laid the foundation for the related research of m7G subsequent modification.

Not only is m7G involved in the normal physiological metabolism of RNA, but recent research also shows that m7G and related regulators appear to be considerably dysregulated in tumors [12]. Here, we review current advances in the biological role of m7G modifications, potential molecular mechanisms of tumorigenesis and prospects for further research.

## Detection method of m7G modification

The detection of RNA modifications is fundamental to the study of m7G modifications in the regulation of gene expression. The main methods include quantitative detection and high-throughput sequencing (Table 1). The former is represented by liquid chromatography-mass spectrometry (LC-MS/MS) and northern blot, which are used to assess the degree of overall m7G modification in tRNA [11, 13, 14]. In addition, the level of m7G modification of rRNA can be detected by primer extension [15]. The aforementioned techniques, however, have limited resolution and can not detect m7G modification sites at single nucleotide base resolution [16].

**Table 1:** Commonly used assays for m7G modification

Technique	Quantitative	Sensitivity	RNA type	Resolution	Main principle	References
Northern blot	yes	low	tRNA	bulk-level RNA	total RNA isolation and RNA blot hybridization	[14]
Primer extension	no	low	rRNA	fragmented RNA	reverse transcription	[15]
LC-MS/MS	yes	low	mRNA, tRNA	bulk-level RNA	liquid chromatography combined with mass spectrometry	[6]
m7G-MERIP-seq	yes	low	mRNA	fragmented RNA	RNA immunoprecipitation	[11]
m7G-miCLIP-seq	yes	high	mRNA	single-base resolution	immunoprecipitation and individual-nucleotide-resolution cross-linking	[4]
m7G-MAP-seq	yes	high	tRNA, rRNA	single-base resolution	chemical reduction and reverse transcription	[18]
m7G-seq	yes	high	mRNA, tRNA	single-base resolution	chemical reduction and reverse transcription	[11]
AlkAniline-seq	yes	high	tRNA, rRNA	single-base resolution	chemical cleavage	[19]
BoRed-seq	yes	high	miRNA	single-base resolution	chemical reduction and reverse transcription	[6]
TRAC-seq	yes	high	tRNA	single-base resolution	chemical cleavage	[17]
m7G-quant-seq	yes	high	tRNA	single-base resolution	chemical reduction and reverse transcription	[20]

High-throughput sequencing refers to the use of antibody immunoprecipitation or chemical methods to accurately locate the site of m7G modification in RNA. Antibody-based analysis mainly includes immunoprecipitation of methylated RNA immuno-precipitation coupled with next-generation sequencing combined with next-generation sequencing (m7G MERIP-Seq) [11, 17] and Immunoprecipitation sequencing (m7G-miCLIPseq) [4]. Both sequencing methods are prone to false positive due to non-specific binding of antibodies [11]. While the former can only detect which mRNAs undergo m7G methylation modification, the latter can identify specific m7G modification sites with single-base resolution [4, 11, 17]. There are two main types of analysis based on chemical detection, one is chemical reduction and reverse transcription methods, such as m7G-MAP-seq [18], m7G-Seq [11]. m7G-Seq utilizes chemical reduction and deamination to selectively convert the m7G site into the basic site, and by reverse transcription enzyme to successfully detect the m7G signal within the mRNA (11). m7G-MAP-seq reduces the m7G site to a basic site by sodium borohydride, which is directly recorded as a cDNA mutation by reverse transcription and sequenced (18). The other is chemical cleavage-mediated detection, such as alkaline hydrolysis and aniline cleavage sequencing (AlkAniline-Seq) [19] and tRNA reduction and cleavage sequencing (TRAC-Seq) [17]. AlkAniline-Seq generates 5’-phosphate bonds by aniline cleavage and is used for library preparation [19]. TRAC-Seq is similar in principle, except that the former uses total RNA as the starting material, while the latter uses small RNA as the starting material and adds AlkB demethylation and sodium borohydride reduction steps, resulting in highly efficient reverse transcription of tRNAs [17]. All four chemical methods mentioned above detect the m7G modification site at single nucleotide resolution, but the accuracy of them remains poor due to partial reduction, deamination and cleavage of the m7G site [11, 20]. Because of this, Zhang et al. further optimized m7G-Seq and developed m7G-quant-seq, which achieved efficient reduction and purification of m7G sites inside tRNA [20].

## m7G modifications in RNA

**Fig. 2:**
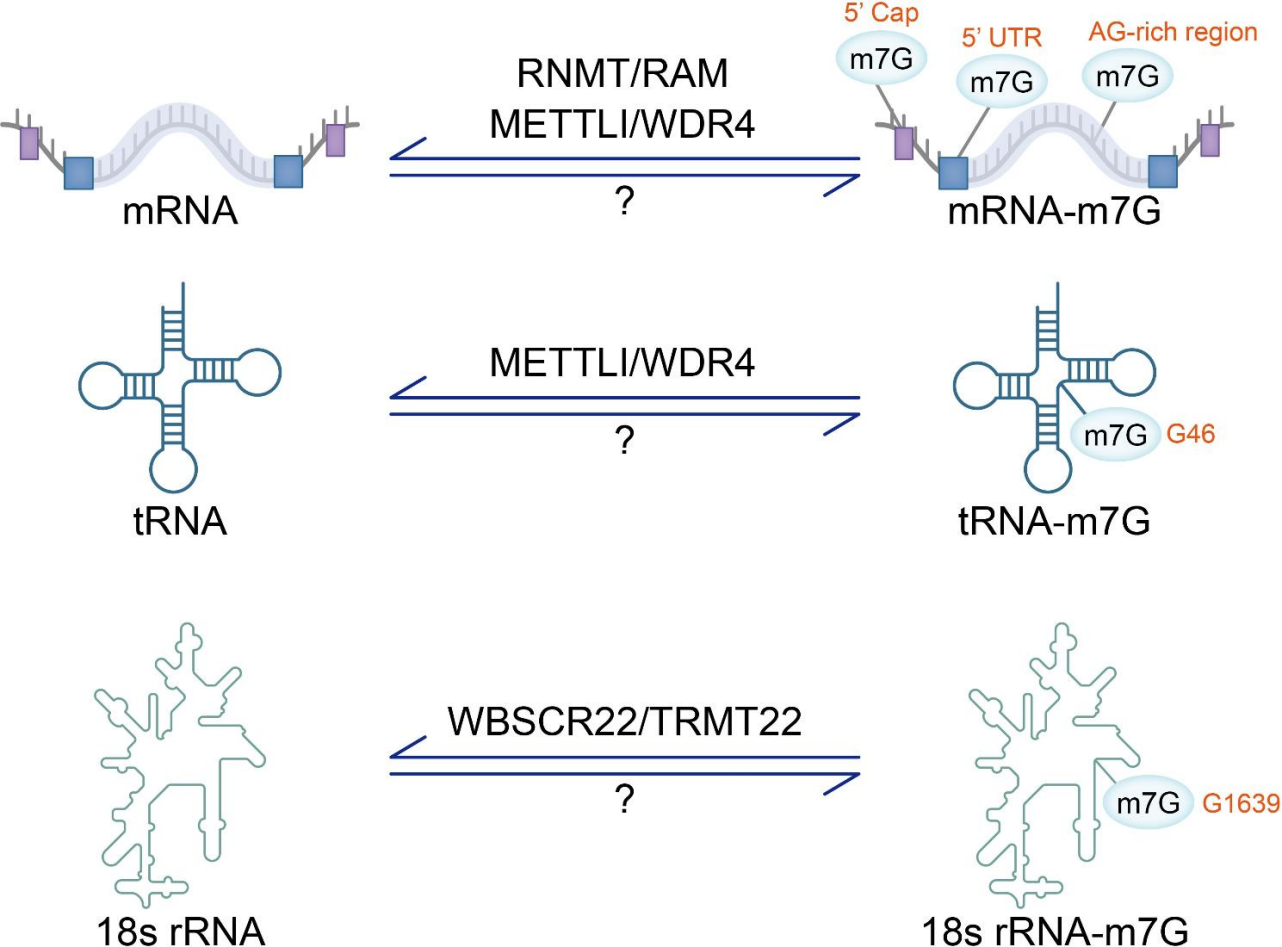
The process and location of m7G modifications in RNA. m7G modifications are present at the 5’cap, 5’UTR and A-G rich regions of mRNA. Where methylation at the 5’cap is mediated by RNMT/RAM, methylation within the mRNA is mediated by METTL1/WDR4. METTL1/WDR4 is the enzyme that catalyzes m7G methylation at the tRNA G46 position. The methyltransferase WBSCR22/TRMT112 catalyzes the 18s rRNA G1639 site

**Fig. 3:**
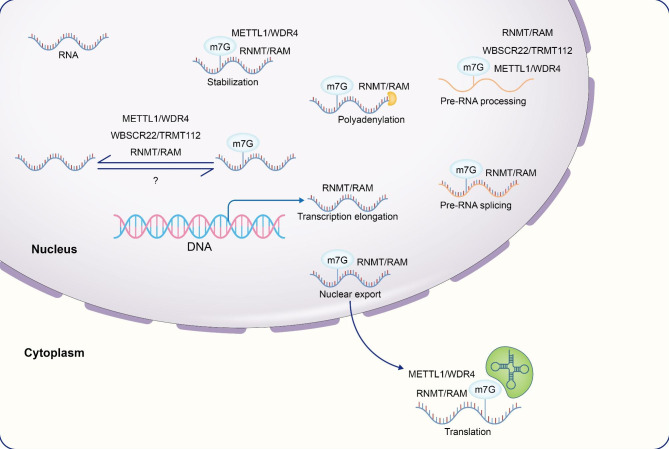
Processes that involve m7G modifications in the metabolism of RNA. m7G regulators are involved in post-transcriptional extension, splicing, polyadenylation, nuclear export, and translation of RNA. They also stabilize the structure of post-transcriptional RNA

In recent years, it has become possible to identify the overall modification level and internal sites of RNA methylation with the continuous improvement of sequencing methods. The m7G modification has been shown to exist in mRNA, tRNA and rRNA (Fig. 2), and plays an important role in normal physiological functions of the human body (Fig. 3).

### m7G modifications in mRNA

The m7G modification of eukaryotic mRNA was first discovered at its 5’ end. The RNMT/RAM methyltransferase complex uses SAM as a methyl donor to form a “cap” structure of m7G(5’)ppp(5’)X at the N7 position of guanine nucleotide [21–23]. The m7G cap binds to the eukaryotic translation initiation factor (elF4E) and participates in the initiation of translation [24]. It can interact with the cap-binding complex to recruit Npl3 and Yra1 and increase the nuclear export of mRNA [25, 26]. It can resist the activity of related RNA hydrolases and stabilizes the structure of mRNA [27].

In addition, there are also many m7G modification sites inside mRNA [28]. Zhang et al. found that the m7G cap was removed, and the proportion of m7G in mammalian mRNA was 0.02%-0.05%. At the same time, based on the method of MERIP-seq and reverse transcription, a new sequencing method—m7G-Seq was developed by using the characteristics of m7G that is prone to chemical reduction reaction, which realized the accurate positioning of m7G modification inside mRNA at base resolution. Using this technique, they found significant enrichment of m7G in mRNA 5’UTRs, coding sequences (CDSs) and 3’UTRs as well as AG-rich environments [11]. Similarly, Malbec et al. found m7G peaks in the AG-rich non-coding region at the 5’ end of mRNA in human and mouse cells through m7G miCLIP-seq. At the same time, they also found that although m7G modification is highly conserved in mammals, m7G modification inside mRNA is dynamically regulated under stress conditions. Once heat shock and oxidative stress occurred, the abundance of m7G modification in mRNA CDSs and 3’UTR regions was significantly increased, the abundance of m7G modification in 5’UTRs was significantly reduced, and the mRNA translation efficiency increased [4]. Considering that 3’UTRs are key regulatory regions for gene expression and translation, it is reasonable to suspect that m7G modification sites in the 3’ UTRs are switches that regulate mRNA translation efficiency.

### m7G modifications in ncRNA

M7G modification not only plays an important role in regulating the translation of mRNAs but also has important implications for the expression and function of ncRNAs.

Currently, m7G modification in tRNA is the most studied. The N7 guanine atom at position 46 of the variable loop is typically methylated by tRNA methyltransferases to generate m7G46 [5]. It interacts with the hydrogen bond of the C13-G22 base pair in the L-type tRNA structure to form a special positively charged M7G46-C13-G22 structure that stabilizes the three-dimensional core of tRNA [29, 30]. m7G46 was first detected in yeast tRNA in 1965, through the formation of m7G modification by Trm8p/Trm82p heterodimer complex [5, 11]. Later, Zhang et al. also confirmed the existence of the m7G modification in mammalian tRNAs using m7G-Seq (11). Similar to mRNA, m7G modification sites in ncRNAs are also significantly enriched in AG sequences [4]. It has been proven that tRNA modifications in the anticodon region are vital for the regulation of translation and contribute to occurrence and development of cancer by improving the translation efficiency of oncogenes [12]. At the same time, once the modification of m7G46 in tRNA is defective, it will lead to neurodevelopment-related diseases [31, 32], indicating that m7G tRNA is necessary for the normal differentiation of neural lineages.

There are also m7G modifications in rRNA [18, 19]. On yeast 18 S rRNA, G1575, located on a ridge between the P-site and E-site tRNA, is methylated by Bud23-Trm112 methyltransferase complex N7, while the human 18 S RNA has a similar structure at the position G1639 [15, 33]. Additionally, it has been demonstrated that the G1405 position on the bacterial 16s RNA is selectively methylated to form the m7G modification, which results in resistance to aminoglycosides [34]. However, the role of m7G modification in rRNA is not fully understood and further exploration is needed.

In addition, it has been noted that m7G modification also occurs in miRNAs. Pandolfini et al. successfully pinpointed m7G modification at the G11 site of a specific subset of miRNAs let-7e-5p by developing borohydride reduction sequencing (BoRed-seq) technology. Here, the m7G encourages the creation of G-quadruplexes in miRNAs and aids in the processing of precursor miRNAs [6]. However, it is worth noting that m7G modification was not detected in miRNAs including human Let-7e utilizing m7G-maP-seq by Enroth et al. [18]. Therefore, more high-throughput sequencing technologies are required to further detect m7G modifications in miRNAs.

## Regulators of m7G

m7G modification and related regulatory factors play an important role in the maintenance of normal physiological functions of the human body and the occurrence of cancer. So far, m7G regulators in mammals include METTL1/WDR4, RNMT/RAM and WBSCR22/TRMT112, all of which are methyltransferases that transfer the active methyl group from the donor to the RNA ribosome at position N7 of bird gan, forming m7G modifications.

### METTL1/WDR4

METTL1 is the most typical RNA m7G methyltransferase, which frequently forms a complex with WDR4 to catalyze methylation reactions [11]. METTL1 is located in the 12q13 region, contains 276 amino acids, and can be folded into 8 α-helices and 7 β-sheets [35, 36]. METTL1 is phosphorylated by PKB and RSK at Ser27 both in vivo and in vitro, losing activity and promoting cell growth [37]. WDR4 is a member of the WD repeat protein family, located in the 21q22.3 region, contains 412 amino acids, can be folded into 4 α-helices and 28 β-sheets, and is a homologue of yeast Trm8p/Trm82p [35]. It was shown that the expression of WDR4 significantly correlated with the level of METTL1 protein [14], indicating that WDR4 is an essential cofactor of METTL1. The METTL1/ WDR4 complex is usually able to introduce m7G modification at the G46 site of the variable loop of tRNA, stabilize and affect the tertiary structure and function of tRNA [38, 39] as well as allowing insulin and associated growth factors to control it [37]. It has been revealed that the METTL1/WDR4 complex is essential for embryonic stem cell self-renewal and differentiation. In mouse embryonic stem cells (mESC), the METTL1 gene is knocked down. On the one hand, this affects the rate of cell division and colony formation. On the other, it interferes with the differentiation of embryonic stem cells into the neural lineage and encourages the differentiation of the endodermal and mesodermal lineages [31, 40]. Similarly, mutations in WDR4 cause a distinctive form of microcephalic primordial dwarfism accompanied by marked facial and cerebral malformations and seizures, which may be driven by a decrease in m7G methylation modifications in tRNA [41]. Not only that, but recent studies have also suggested a strong correlation between lower METTL1/WDR4 and neurological disorders such as Down syndrome [42], multiple sclerosis [43], cerebral ischemia [44] and Alzheimer’s disease [45]. The specific mechanism awaits further elucidation in the future.

Meanwhile, the METTL1/ WDR4 complex is able to influence the effectiveness of mRNA translation by altering tRNA m7G modifications. It has been shown that silencing METTL1 impairs m7G tRNA modification, resulting in increased ribosome suspension at the tRNA binding site (site A) and blocked ribosome translocation, thereby reducing protein abundance and decreasing the overall translation efficiency of intracellular mRNAs [12, 31, 46]. Conversely, increased mRNA translation efficiency due to METTL1 upregulation is inextricably linked to cancer development. It has been shown that upregulation of METTL1 increases methylation modification levels of m7G tRNA (especially Arg-TCT tRNA), which reduces ribosomal pausing at the AGA codon and promotes the efficiency of translation of mRNA associated with regulation of the cell cycle and oncogenic mRNA, leading to cancer development and progression [47].

In addition, METTL1 can also promote post-ischemic angiogenesis. It was shown that METTL1 was able to increase the translation of VEGFA mRNA in an m7G modification-dependent manner and promote the proliferation and migration of vascular endothelial cells [48]. This could emerge as a new therapeutic target for ischemic brain diseases.

### WBSCR22/TRMT112

WBSCR22/TRMT112, as a functional homolog of yeast Bud23-Trm112, has been shown to be an m7G methyltransferase complex involved in m7G methylation modification of 18 S rRNA [33, 49, 50]. WBSCR22 was originally identified as one of 26 genes delated in Williams syndrome and contains a nuclear localization signal and a highly conserved S-adenosyl-L-methionine binding motif [51, 52]. It is currently believed that WBSCR22 is involved in the processes of organ regeneration and wound healing [53], enhances glucocorticoid receptor function [54], and regulates lung inflammation and is associated with cancer development and drug resistance [55–57]. A tiny, evolutionarily conserved protein called TRMT112 participates in rRNA m7G modifications by working as a cofactor for WBSCR22 [58]. It turns out that TRMT122 is essential for the metabolic stability of WBSCR22 and the two assemble into a heterodimeric methyltransferase complex that helps pre-rRNA processing to synthesize 18 S rRNA and install the m7G modification at the G1639 position of 18 S rRNA [15].

Interestingly, pre-rRNA processing does not require the catalytic activity of WBSCR22 methyltransferase. It was established that mutants that substituted amino acids on key functional residues of WBSCR22 methyltransferase had no impact on the processing of pre-rRNA. However, if the WBSCR22 gene is silenced, the amount of mature 18 S rRNA will be significantly reduced [15]. It suggests that methyltransferase can aid in the methylation modification of mature rRNA after pre-rRNA processing, independent of its catalytic role. The function of the 18 S rRNA m7G1639 is still unclear. However, it is certain that its high degree of conservation has an important impact on the translation process of ribosomes.

### RNMT/RAM

Human RNMT, also a regulator of m7G modification, is a nuclear protein consisting of 476 amino acids [59, 60]. There can be interaction between RNMT and T cells. RNMT is a key mediator of T cell activation induced by TCR stimulation. At the same time, activated T cells also help RNMT to synthesize mRNA, rRNA, and snoRNA by m7G cap, specifically regulate ribosome abundance and improve the efficiency of translation [61]. RAM is the subunit activated by RNMT and the two bind with each other to form a stable complex structure that aids in the introduction of m7G at the 5’ cap end of mRNA modification and promotes mRNA maturation [60, 62]. In addition, RAM facilitates the recruitment of methyl donors, augments RNMT methyltransferase activity, and regulates m7G modification as well as the expression of related genes at the mRNA cap [21, 60, 63].

## Abnormalities of m7G in cancer

**Fig. 4:**
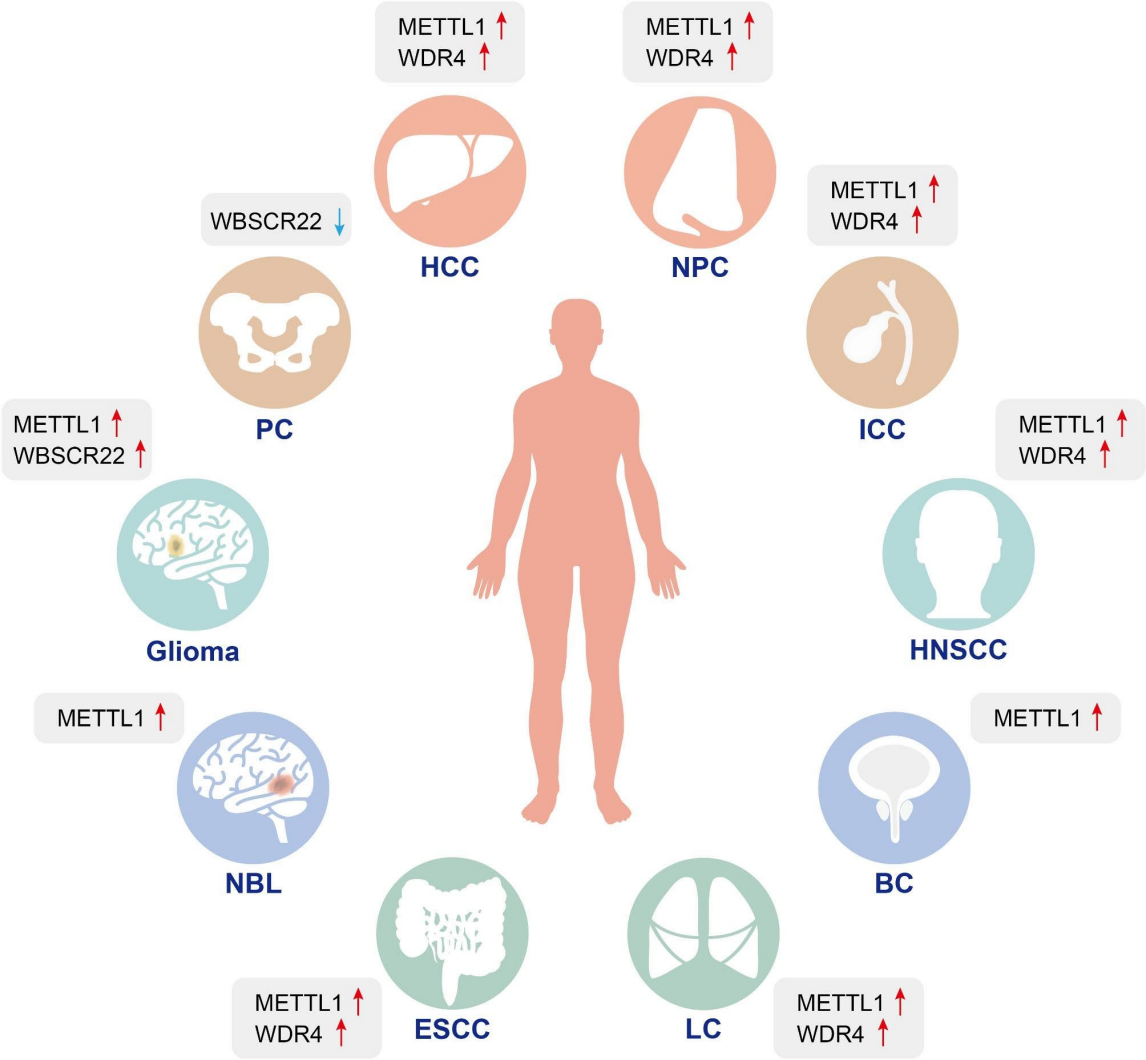
Aberrant expression of m7G modifications in various tumors. m7G regulators act as accelerators to promote the proliferation and progression of HCC, NPC, ICC, HNSCC, BC, LC, ESCC, NBL, Glioma, and as suppressors to inhibit the development of PC. HCC: Hepatocellular carcinoma; NPC: Nasopharyngeal carcinoma; ICC: Intrahepatic cholangiocarcinoma; HNSCC: Head and neck squamous cell carcinoma; LC: Lung cancer; ESCC: Esophageal squamous cell carcinoma; NBL: Neuroblastoma; BC: Bladder Cancer; PC: Pancreatic cancer

**Fig. 5:**
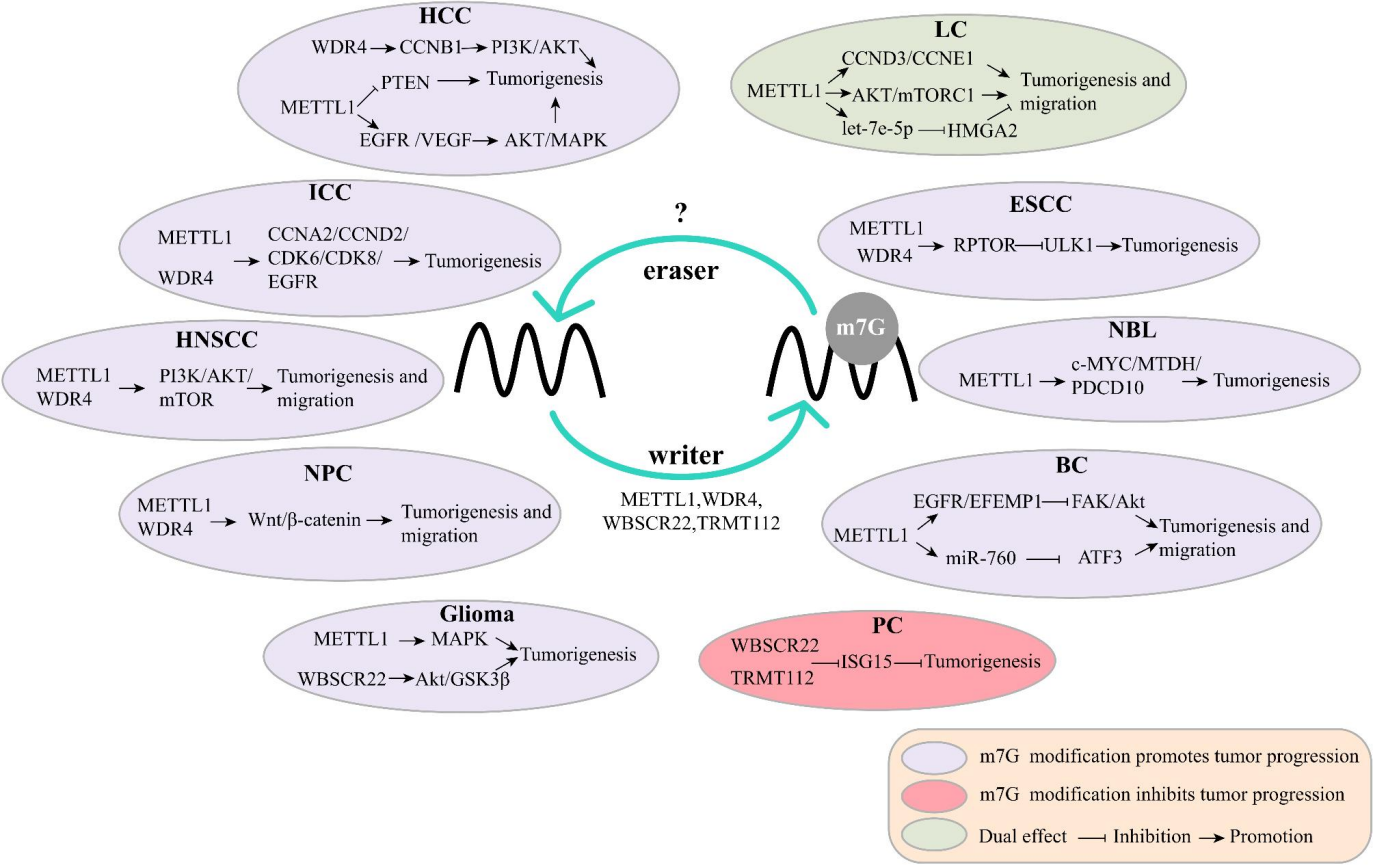
Regulatory mechanisms of m7G modifications in cancer development

Although the current research on m7G modification is not sufficient, more and more evidence shows that m7G modification is involved in the mechanism of cancer development and is related to drug resistance [12, 47]. Here, we summarize the role of m7G modifications in a variety of the most common cancers (Fig. 4) and the associated mechanisms (Fig. 5).

### Liver cancer

Liver cancer is the fifth most common cancer in the world, the second and fourth leading cause of cancer-related death in men and women, and has a highly poor prognosis [64, 65]. Primary liver cancer mainly includes hepatocellular carcinoma (HCC) and intrahepatic cholangiocarcinoma (ICC). The former originates from the parenchymal cells of the liver and accounts for 80% of hepatocellular carcinoma incidence. The latter originates from bile duct epithelial cells and is more malignant than HCC and its three-year survival rate is only 30% [66–68].

It was shown that m7G tRNA modification and its methyltransferases METTL1, WDR4 and WBSCR22 were significantly elevated in HCC and promoted the proliferation, migration and invasion of hepatocellular carcinoma cells [46, 69]. In HCC, knockdown of METTL1 resulted in reduced tRNA m7G modification, severely impaired mRNA translation efficiency, and decreased Cyclin A2, EGFR, and VEGFA protein expression levels. At the same time, the activity of downstream signaling pathways Akt and MAPK of EGFR and VEGFA decreased, which made the cell cycle arrest and inhibited the progress of HCC [46]. Furthermore, METTL1 can also promote the progression of HCC through the downregulation of PTEN pathway activity [70], suggesting that METTL1 could play a regulatory role in the development of hepatocellular carcinoma through multiple pathways. Meanwhile, WDR4 promotes the G2/M cell cycle transition leading to cell proliferation. And by promoting the binding of EIF2A to CCNB1 mRNA, the translation efficiency of CCNB1 rises, activating the PI3K/AKT signaling pathway and assisting in the transformation of EMT. Moreover, CCNB1 can ubiquitinate p53 and degrade p53, which promotes carcinogenesis [13]. At present, the primary mechanism through which WBSCR22 regulates tumors is currently under investigation but must be further elaborated. However, there is no doubt that METTL1, WDR4 and WBSCR22 are potential targets for the future treatment of hepatocellular carcinoma.

In addition, the METTL1/WDR4 complex was also significantly upregulated in ICC. The total levels of mRNA translation were decreased by METTL1 knockdown because it caused a reduction in the frequency of m7G-tRNA-dependent decoding codons and an increase in ribosomal pauses. Interestingly, it was discovered that knockdown of METTL1 did not alter mRNA levels of cancer-related genes, but its translation efficiency was impaired, leading to a drop in cell cycle-related proteins such as CCNA2, CCND2, CDK6, CDK8 and EGFR, which affected tumor progression [71].

### Head and neck squamous cell carcinoma

Head and neck squamous cell carcinoma (HNSCC), which originates from mucosal epithelial cells of the oral cavity, pharynx, and larynx, is the sixth most prevalent cancer worldwide [72, 73]. Upregulation of the METTL1/WDR4 complex promotes tumor development and metastasis. Mechanistically, the decrease of METTL1/WDR4 complex reduces the m7G level of tRNA and inhibits the translation of mRNA involved in PI3K/AKT/mTOR-related pathway genes. Due to the decreased phosphorylation level of PI3K/AKT/mTOR pathway, the expression of its downstream related proteins including Cyclin D1, Vimentin, MMP9, Bcl-2 and P-S6K decreased, and the expression of tumor suppressor gene bax increased [74]. Meanwhile, it was shown that the phosphorylation of the PI3K/AKT/mTOR pathway mediated by METTL1 is necessary for the development of HNSCC, and targeting METTL1 will become an important therapeutic target in the future.

Although nasopharyngeal carcinoma is a type of HNSCC, its epidemiology and pathogenesis are not exactly the same as those of HNSCC [75]. In NPC, upregulation of METTL1/WDR4 increases the expression of m7G tRNA, and through upregulation of WNT/β-catenin signaling pathway, it increases EMT transformation and promotes carcinogenesis [76].

### Bladder cancer

Bladder cancer (BC) is the ninth most common malignancy worldwide, and uroepithelial carcinoma is its main pathological type [77, 78]. In recent years, it has been discovered that increased METTL1 is positively related to the emergence of BC. Mechanistically, Mechanistically, METTL1 increases the decoding frequency of tRNA by promoting the m7G modification of tRNA, which increases the translation efficiency of EGFR/EFEMP1, and inhibits its signal transduction of its downstream signaling pathway FAK/Akt [79]. Another study demonstrated that METTL1 affected BC progression through the miR-760 axis. METTL1 facilitated pre miR-760 processing in an m7G-dependent manner and helped miR-760 maturation. The researchers observed that increased miR-760 will downregulate oncogenic protein AFT3, proving that METTL1 regulates BC advancement through the m7G-miR-760/ATF3 axis. Since miRNAs tend to bind to the 3’ non-coding regions of their targeted mRNAs, silencing mRNA expression [80]. METTL1 may become an important potential target for BC therapy.

### Lung cancer

In recent years, the incidence of lung cancer has gradually increased and the mortality rate ranks first in the world [81]. Despite breakthroughs have been made in targeted therapy for lung cancer, the five-year survival rate is just less than 20% [82]. METTL1 and WDR4 were found to be up-regulated in lung cancer tissues which promoted tumor cell proliferation and migration. Loss of METTL1 reduces the expression of m7G tRNA, resulting in decreased translation efficiency of cell cycle regulators CCND3 and CCNE1 mRNA [14]. In addition, METTL1 inhibits the proliferation and autophagy of A549 cells through the AKT/mTORC1 signaling pathway [83]. Paradoxically, however, Pandolfifini et al. discovered that METTL1 reduced lung cancer migration by encouraging the processing of m7G miRNAs. Additionally, METTL1 inhibits the proliferation and autophagy of A549 cells through the AKT/mTORC1 signaling pathway [6]. This may be caused by miRNAs which inhibit their target mRNAs from being able to translate. METTL1 may have both positive and negative effects on the emergence of lung cancer.

### Esophageal cancer

Esophageal cancer is the eighth most common malignancy in the world, mainly divided into esophageal squamous cell carcinoma (ESCC) and esophageal adenocarcinoma [84, 85]. ESCC is the major subtype of esophageal cancer in Asia [86]. It turns out that high expression of METTL1 and WDR4 was positively correlated with poor prognosis of ESCC. Knockdown of METTL1 caused the ribosome to pause at the codon decoded by m7G tRNA, which significantly reduced the translation efficiency of RPTOR (regulatory-related protein of mTOR complex 1) and improved the phosphorylation level of its downstream target gene ULK1, leading to increased cell death and autophagy in ESCC cells and slowing down the migration and progress of ESCC [87, 88]. METTL1/ RPTOR/ ULK1 autophagy axis is a vital target for the future treatment of ESCC.

### Neuroblastoma

Neuroblastoma (NBL) is a neuroendocrine tumor originating from the sympathetic nervous system [89]. Although of low incidence, it is the most common extracranial solid cancer in children [90]. Due to its low survival rate, it is extremely important to develop new therapeutic strategies. It was found that METTL1 overexpression in NBL is a distinct risk factor for poor prognosis. By boosting the expression of m7G tRNA, METTL1 improves the efficiency of mRNA translation of c-MYC transcriptionally activated genomic and cell cycle, which includes the common oncogenes metadherin (MTDH) and programmed cell death 10 (PDCD10) [91].

### Glioma

Glioma is a primary brain tumor derived from glial cells. It is very malignant and is categorized by WTO as low-grade glioma(LGG) and high-grade glioma(HGG) [92]. Both METTL1 and WBSCR22 are reported to be highly expressed in gliomas [57, 93]. The glioma grade-dependent increase in METTL1 suggests that METTL1 considerably reinforces the growth of tumor cells. It’s possible that MELLL1’s stimulation of the downstream MAPK signaling pathway is the cause of this [93]. WBSCR22 promotes the proliferation of tumor cells by phosphorylating Akt and GSK3 and raising the levels of β-catenins and CyclinD1 in glioma cells [57].

### Pancreatic cancer

Pancreatic cancer is a highly malignant tumor of digestive tract. The five-year survival rate is 7% and the incidence rate is almost equal to mortality [94]. Understanding its pathophysiology is crucial due to the extremely bad prognosis. WBSCR22 has been reported to inhibit the proliferation and migration of tumor cells, and TRMT112 as its cofactor can enhance the effect of WBSCR22 as a tumor suppressor. Mechanistically, high expression of WBSCR22 can downregulate the translation of the oncogenic factor interferon-stimulated gene 15 (ISG15) and reverse the oncogenic effect of ISG15 [56].

## Roles of m7G modification in the tumor immune microenvironment

In recent years, the role of tumor microenvironment (TME) in the development of tumors has attracted great attention. TME, which is strongly related to tumor development, immune evasion, immune tolerance, and drug resistance [95], is the internal environment in which tumor cells, cancer-associated fibroblasts (CAF), endothelial cells, adipocytes, and other types of immune cells develop [96, 97].

More and more studies have found that m7G modification and its related regulatory regulators play an important role in TME [98, 99]. Promyelocytic leukemia protein (PML) is a tumor suppressor protein that plays an important role in TME [100]. In lung cancer, WDR4 degrades PML through the ubiquitination pathway and upregulates its downstream CD73, urokinase plasminogen activator receptor (uPAR) and serum amyloid A2 (SAA2) via HIF-1. These three proteins could establish a pro-metastatic tumor microenvironment through multiple mechanisms. Meanwhile, the WDR4/PML axis can reduce CD8 + T cells and increase Treg and M2-like macrophages in TME [100]. M2 macrophages are a unique subpopulation derived from monocytes. Unlike M1, they can secrete a variety of immunosuppressive cytokines such as IL-10 and TGF-β to produce immunosuppression [101]. Treg cells are mainly derived from the thymus and have immunosuppressive effects [102]. TME inhibits the immune response of CD8 + T cells by inducing the expression of PD-1 in tumor-infiltrating Treg cells, thereby enabling tumor cell immune evasion [103]. In HNSCC, knockdown of METTL1 altered both the composition of immune cells in the TME and how they communicate with tumor cells. In METTL1 knockout mice, CD4 + T and CD8 + T were significantly upregulated, and Treg and Th17 were significantly reduced. Moreover, interleukin 1 (IL1b)-interleukin 1 receptor 2 (IL1r2) and IL1b- interleukin 1 receptor 1 (IL1r1) suppressed the interstitial and epithelial cell receptor-ligand carcinogenic pathways [74]. In adrenocortical carcinoma, immunofluorescence revealed that high expression of METTL1 in tumor cells was inversely proportional to CD8 + T and directly proportional to the infiltration rate of macrophages [98]. Numerous bioinformatic analyses have also demonstrated that m7G modification related regulators, as well as lncRNAs, have important impact on TME [98, 104, 105].

## m7G modification and tumor drug resistance

Although numerous drugs have been developed to treat cancer, their resistance to treatment and low patient survival rates remain frustrating. In recent years, an increasing number of studies have revealed the association of m7G modifications and related regulators with cancer drug resistance, as summarized below (Table 2). By regulating the miR149-3p/S100A4/P53 axis in colon cancer (CC), increased METTL1 promotes the lethal effect of cisplatin on colon cancer cells [106]. In contrast, overexpression of WBSCR22 reduces oxaliplatin-induced intracellular reactive oxygen species (ROS) and 8-oxoguanine (8-oxoG) production in colon cancer cells, leading to decreased sensitivity to oxaliplatin treatment [55]. In hepatocellular carcinoma, the METTL1/WDR4 complex promoted the translation of EGFR pathway genes via modulating m7G tRNA modification, which decreased the tumor’s susceptibility to Lenvatinib [107]. Meanwhile, knockdown of METTL1 could improve the sensitivity of Hela cells to 5-fluorouracil [108]. In addition, METTL1 and WDR4 drive drug resistance by altering the tumor microenvironment. Previous studies have indicated that TME is associated with drug resistance, probably because of the changed dynamics of TME, which enable the signaling pathways that chemotherapeutic agents target to change and lose their initial lethal effect [95]. In nasopharyngeal carcinoma, METTL1 increases the conversion of EMT through the WNT/β- catenin signaling pathway, leading to chemoresistance to cisplatin and docetaxel in vitro and in vivo [76]. Likewise, WDR4 also reduced the sensitivity of hepatocellular carcinoma cells to sorafenib by enhancing the translation of CCNB1 and the conversion of EMT [13]. This illustrates the significant role that METTL1 and WDR4 play in mediating cancer drug resistance and is expected to be a future therapeutic target.

**Table 2:** Drug resistance in cancer mediated by m7G regulators

m7G regulators	Tumors	Drug	Function	mechanism	References
METTL1	Colon cancer	Cisplatin	Increase drug sensitivity	Regulate miR149-3p/S100A4/P53 axis	[106]
METTL1, WDR4	Hepatocellular carcinoma	Lenvatinib	Decrease drug sensitivity	Promote the translation of EGFR	[107]
METTL1	Nasopharyngeal carcinoma	Cisplatin and Docetaxel	Decrease drug sensitivity	Activate WNT/β- catenins/EMT pathway	[76]
WDR4	Hepatocellular carcinoma	Sorafenib	Decrease drug sensitivity	Promote the translation of CCNB1	[11]
METTL1	Cervical cancer	5-Fluorouracil	Decrease drug sensitivity	Lead to rapid tRNA(Val^AAC^) decay	[108]
WBSCR22	Colon cancer	oxaliplatin	Decrease drug sensitivity	Reduce intracellular ROS and 8-oxoG	[55]

## Conclusions and future direction

This paper reviews the role of m7G modifications and related methyltransferases in regulating RNA metabolic processes including transcription, splicing, nuclear export, translation and related biological processes, and describes the mechanisms involved in the regulation of cancer development by m7G modifications, which suggests that m7G modifications and their regulators are important targets for intervention in future cancer therapy.

The expression of METTL1/WDR4 is abnormally elevated in most carcinomas, and by enhancing m7G tRNA expression, ribosome suspension is reduced, the efficiency of associated oncogenes’ mRNA translation is boosted, and downstream oncogenic signaling pathways like PI3K/AKT and MAPK are activated [12]. Tumor suppressor genes is also declining in the meantime. M7G methylation, however, is a dual regulator of tumor growth. Upregulation of METTL1 demonstrates suppressive effects in teratoma with CC [40, 106] but has considerable pro-oncogenic effects in HCC, ICC, HNSCC, NPC, BC, LC, ESCC, NBL, and glioma [14, 46, 71, 74, 76, 79, 80, 87, 91, 93]. The same is true for WBCCR22, which both suppresses PC and promotes glioma [56, 57]. In addition, m7G modification has a contradictory dual role in the same tumor. In lung cancer, METTL1 downregulates the production of oncogenes through miRNAs [6] while simultaneously promoting tumor cell proliferation through the AKT/mTORC1 signaling pathway [14]. This shows that METTL1 affects lung cancer both positively and negatively, but ultimately exhibits cancer-promoting effects through a complex mechanism.

The mRNA 5’ end is not only modified by m7G. In fact, it contains combinatorial RNA modifications, including m6A, m6Am and Am. They are located close to each other and can influence each other [109]. A dynamic and reversible epitranscriptomic modification is produced when Am is typically transformed to m6Am in a m7G cap-dependent fashion by the methyltransferase Phosphorylated CTD interaction factor 1 (PCIF1). From there, m6Am can be converted back to Am in an m7G modification-dependent manner by the demethylase FTO [109–112]. This suggests that post-transcriptional modifications are part of a complex network of institutions that regulate physiological processes in the body rather than acting alone. However, in addition to the dynamic association with m6Am modification, whether m7G can also cooperate with other modifications needs further exploration and research.

In addition to being involved in cancer drug resistance, M7G modifications also mediate cancer resistance to other treatments. Radiation resistance in HCC is caused by increased expression of METTL1. This is mainly because, following radiation, METTL1 improves translation of the DNA-dependent protein kinase catalytic subunit or DNA ligase IV, which in turn accelerates non-homologous end joining (NHEJ)-mediated DNA double-strand break repair [113]. Furthermore, METTL1 modulates the downstream SLUG/SNAIL signaling pathway via m7G tRNA for HCC tissues following radiofrequency ablation to enhance the malignant potential of HCC [114]. Perhaps conventional cancer treatment paired with specific METTL1 inhibitors can achieve better therapeutic effect.

At present, there are still a lot of limitations in the study of m7G modification and related regulators. Since m7G is a newly discovered RNA modification in recent years, demethylases (eraser), methylated reading proteins (reader) and other methyltransferases (writer) involved in this process have not been identified yet. In addition, due to the creation of the m7G database [115], researchers have predicted some regulators associated with m7G modification, but further experimental proof is still required to determine whether they are indeed involved in the formation of m7G modification.

At the same time, due to technical limitations, there is still debate on whether there are m7G methylation sites inside miRNA [4, 6]. therefore, new high-resolution sequencing tools urgently need to be developed to help us better understand the m7G modification profiles inside RNAs. In recent years, the m7G sites inside RNAs have been simulated by several web servers, including iRNA-m7G [116], XG-m7G [117], m7G-IFL [118], m7GFinder [115], m7G-DPP [119] and m7GPredictor [120]. This will help us to update the internal m7G sites faster.

Future studies will concentrate on the function of m7G modification in TME, which will help us improve the effectiveness of cancer immunotherapy’s response rate. It is well known that M2 macrophages, myeloid-derived suppressor cells (MDSCs) and Treg cells are the main immunosuppressive cells that restrict the immune response by secreting a variety of suppressors in TME [95]. Encouragingly, Liu et al. have demonstrated that targeting METTL1-mediated tRNA modification reduces polymorphonuclear myeloid-derived suppressor cells (PMN-MDSCs) and improves anti-PD-1 efficacy in a mouse model of ICC [121]. The inhibition of IL1b-IL1R1 signaling in HNSCC was previously mentioned [74]. Recently, Mair et al. demonstrated that Treg was significantly increased in the HNSCC tumor microenvironment. Tumor-infiltrating Treg cells selectively expressed IL1R1 receptors marked a highly suppressed and enlarged fraction of Treg cells and suppressed CD8 + T cells more efficiently in contrast to IL1R1-Treg [122]. This may provide us an idea to think that the HNSCC tumor microenvironment is governed by the m7G modification through this mechanism, but further experiments are still needed to prove it in the future. To date, there are no targeted inhibitors of m7G modification. In view of the great potential of m7G modification in cancer treatment, the emergence of related drugs is expected to improve the effectiveness of targeted drug combined with immunotherapy in the future. In conclusion, m7G modification provides new insights into the mechanism of cancer development and plays an important role in the diagnosis, prognosis, and treatment of cancer, which deserves further investigation.

## Data Availability

Not applicable.

## References

[1] Wiener D, Schwartz S (2021). The epitranscriptome beyond m6A. Nat Rev Genet.

[2] Barbieri I, Kouzarides T (2020). Role of RNA modifications in cancer. Nat Rev Cancer.

[3] Komal S, Zhang L-R, Han S-N (2021). Potential regulatory role of epigenetic RNA methylation in cardiovascular diseases. Biomed Pharmacother.

[4] Malbec L, Zhang T, Chen Y-S, Zhang Y, Sun B-F, Shi B-Y (2019). Dynamic methylome of internal mRNA N7-methylguanosine and its regulatory role in translation. Cell Res.

[5] Tomikawa C (2018). 7-Methylguanosine modifications in transfer RNA (tRNA). Int J Mol Sci.

[6] Pandolfini L, Barbieri I, Bannister AJ, Hendrick A, Andrews B, Webster N (2019). METTL1 promotes let-7 MicroRNA Processing via m7G methylation. Mol Cell.

[7] Lewis JD, Izaurralde E (1997). The role of the cap structure in RNA processing and nuclear export. Eur J Biochem.

[8] Muthukrishnan S, Both GW, Furuichi Y, Shatkin AJ (1975). 5’-Terminal 7-methylguanosine in eukaryotic mRNA is required for translation. Nature.

[9] Pei Y, Shuman S (2002). Interactions between fission yeast mRNA capping enzymes and elongation factor Spt5. J Biol Chem.

[10] Lindstrom DL, Squazzo SL, Muster N, Burckin TA, Wachter KC, Emigh CA (2003). Dual roles for Spt5 in pre-mRNA processing and transcription elongation revealed by identification of Spt5-associated proteins. Mol Cell Biol.

[11] Zhang L-S, Liu C, Ma H, Dai Q, Sun H-L, Luo G et al. Transcriptome-wide mapping of Internal N7-Methylguanosine methylome in mammalian mRNA. Mol Cell. 2019;74(6). 10.1016/j.molcel.2019.03.036 PMC6588483 31031084

[12] Katsara O, Schneider RJ (2021). m7G tRNA modification reveals new secrets in the translational regulation of cancer development. Mol Cell.

[13] Xia P, Zhang H, Xu K, Jiang X, Gao M, Wang G (2021). MYC-targeted WDR4 promotes proliferation, metastasis, and sorafenib resistance by inducing CCNB1 translation in hepatocellular carcinoma. Cell Death Dis.

[14] Ma J, Han H, Huang Y, Yang C, Zheng S, Cai T (2021). METTL1/WDR4-mediated m7G tRNA modifications and m7G codon usage promote mRNA translation and lung cancer progression. Mol Therapy: J Am Soc Gene Therapy.

[15] Zorbas C, Nicolas E, Wacheul L, Huvelle E, Heurgué-Hamard V, Lafontaine DLJ (2015). The human 18S rRNA base methyltransferases DIMT1L and WBSCR22-TRMT112 but not rRNA modification are required for ribosome biogenesis. Mol Biol Cell.

[16] Xia X, Wang Y, Zheng JC (2023). Internal m7G methylation: a novel epitranscriptomic contributor in brain development and diseases. Mol Ther Nucleic Acids.

[17] Lin S, Liu Q, Jiang Y-Z, Gregory RI (2019). Nucleotide resolution profiling of m7G tRNA modification by TRAC-Seq. Nat Protoc.

[18] Enroth C, Poulsen LD, Iversen S, Kirpekar F, Albrechtsen A, Vinther J (2019). Detection of internal N7-methylguanosine (m7G) RNA modifications by mutational profiling sequencing. Nucleic Acids Res.

[19] Marchand V, Ayadi L, Ernst FGM, Hertler J, Bourguignon-Igel V, Galvanin A (2018). AlkAniline-Seq: profiling of m7 G and m3 C RNA modifications at single Nucleotide Resolution. Angew Chem Int Ed Engl.

[20] Zhang L-S, Ju C-W, Liu C, Wei J, Dai Q, Chen L (2022). m7G-quant-seq: quantitative detection of RNA internal N7-Methylguanosine. ACS Chem Biol.

[21] Shatkin AJ (1976). Capping of eucaryotic mRNAs. Cell.

[22] Mandal SS, Chu C, Wada T, Handa H, Shatkin AJ, Reinberg D (2004). Functional interactions of RNA-capping enzyme with factors that positively and negatively regulate promoter escape by RNA polymerase II. Proc Natl Acad Sci U S A.

[23] Pillutla RC, Yue Z, Maldonado E, Shatkin AJ (1998). Recombinant human mRNA cap methyltransferase binds capping enzyme/RNA polymerase IIo complexes. J Biol Chem.

[24] Hayek H, Eriani G, Allmang C. eIF3 interacts with selenoprotein mRNAs. Biomolecules. 2022;12(9). 10.3390/biom12091268 PMC9496622 36139107

[25] Lei EP, Krebber H, Silver PA (2001). Messenger RNAs are recruited for nuclear export during transcription. Genes Dev.

[26] Sen R, Barman P, Kaja A, Ferdoush J, Lahudkar S, Roy A et al. Distinct functions of the Cap-Binding complex in Stimulation of Nuclear mRNA export. Mol Cell Biol. 2019;39(8). 10.1128/MCB.00540-18 PMC6447411 30745412

[27] Ramanathan A, Robb GB, Chan S-H (2016). mRNA capping: biological functions and applications. Nucleic Acids Res.

[28] You X-J, Yuan B-F (2021). Detecting Internal N7-Methylguanosine mRNA modifications by Differential enzymatic digestion coupled with Mass Spectrometry Analysis. Methods Mol Biol.

[29] Kim SH, Sussman JL, Suddath FL, Quigley GJ, McPherson A, Wang AH (1974). The general structure of transfer RNA molecules. Proc Natl Acad Sci U S A.

[30] Rich A, RajBhandary UL, Transfer RNA (1976). Molecular structure, sequence, and properties. Annu Rev Biochem.

[31] Lin S, Liu Q, Lelyveld VS, Choe J, Szostak JW, Gregory RI. Mettl1/Wdr4-Mediated mG tRNA methylome is required for normal mRNA translation and embryonic stem cell Self-Renewal and differentiation. Mol Cell. 2018;71(2). 10.1016/j.molcel.2018.06.001 PMC6086580 29983320

[32] Sauna ZE, Kimchi-Sarfaty C (2011). Understanding the contribution of synonymous mutations to human disease. Nat Rev Genet.

[33] Haag S, Kretschmer J, Bohnsack MT (2015). WBSCR22/Merm1 is required for late nuclear pre-ribosomal RNA processing and mediates N7-methylation of G1639 in human 18S rRNA. RNA.

[34] Husain N, Tkaczuk KL, Tulsidas SR, Kaminska KH, Cubrilo S, Maravić-Vlahovicek G (2010). Structural basis for the methylation of G1405 in 16S rRNA by aminoglycoside resistance methyltransferase Sgm from an antibiotic producer: a diversity of active sites in m7G methyltransferases. Nucleic Acids Res.

[35] Luo Y, Yao Y, Wu P, Zi X, Sun N, He J (2022). The potential role of N7-methylguanosine (m7G) in cancer. J Hematol Oncol.

[36] Bahr A, Hankeln T, Fiedler T, Hegemann J, Schmidt ER (1999). Molecular analysis of METTL1, a novel human methyltransferase-like gene with a high degree of phylogenetic conservation. Genomics.

[37] Cartlidge RA, Knebel A, Peggie M, Alexandrov A, Phizicky EM, Cohen P (2005). The tRNA methylase METTL1 is phosphorylated and inactivated by PKB and RSK in vitro and in cells. EMBO J.

[38] Shi H, Moore PB (2000). The crystal structure of yeast phenylalanine tRNA at 1.93 a resolution: a classic structure revisited. RNA.

[39] Oliva R, Cavallo L, Tramontano A (2006). Accurate energies of hydrogen bonded nucleic acid base pairs and triplets in tRNA tertiary interactions. Nucleic Acids Res.

[40] Deng Y, Zhou Z, Ji W, Lin S, Wang M (2020). METTL1-mediated m7G methylation maintains pluripotency in human stem cells and limits mesoderm differentiation and vascular development. Stem Cell Res Ther.

[41] Shaheen R, Abdel-Salam GMH, Guy MP, Alomar R, Abdel-Hamid MS, Afifi HH (2015). Mutation in WDR4 impairs tRNA m(7)G46 methylation and causes a distinct form of microcephalic primordial dwarfism. Genome Biol.

[42] Michaud J, Kudoh J, Berry A, Bonne-Tamir B, Lalioti MD, Rossier C (2000). Isolation and characterization of a human chromosome 21q22.3 gene (WDR4) and its mouse homologue that code for a WD-repeat protein. Genomics.

[43] Alcina A, Fedetz M, Fernández O, Saiz A, Izquierdo G, Lucas M (2013). Identification of a functional variant in the KIF5A-CYP27B1-METTL1-FAM119B locus associated with multiple sclerosis. J Med Genet.

[44] Androvic P, Kirdajova D, Tureckova J, Zucha D, Rohlova E, Abaffy P (2020). Decoding the Transcriptional response to ischemic stroke in young and aged mouse brain. Cell Rep.

[45] Srinivasan K, Friedman BA, Etxeberria A, Huntley MA, van der Brug MP, Foreman O (2020). Alzheimer’s patient microglia exhibit enhanced aging and unique transcriptional activation. Cell Rep.

[46] Chen Z, Zhu W, Zhu S, Sun K, Liao J, Liu H (2021). METTL1 promotes hepatocarcinogenesis via m7 G tRNA modification-dependent translation control. Clin Translational Med.

[47] Orellana EA, Liu Q, Yankova E, Pirouz M, De Braekeleer E, Zhang W (2021). METTL1-mediated m(7)G modification of Arg-TCT tRNA drives oncogenic transformation. Mol Cell.

[48] Zhao Y, Kong L, Pei Z, Li F, Li C, Sun X (2021). m7G methyltransferase METTL1 promotes post-ischemic angiogenesis via promoting VEGFA mRNA translation. Front Cell Dev Biol.

[49] White J, Li Z, Sardana R, Bujnicki JM, Marcotte EM, Johnson AW (2008). Bud23 methylates G1575 of 18S rRNA and is required for efficient nuclear export of pre-40S subunits. Mol Cell Biol.

[50] Létoquart J, Huvelle E, Wacheul L, Bourgeois G, Zorbas C, Graille M (2014). Structural and functional studies of Bud23-Trm112 reveal 18S rRNA N7-G1575 methylation occurs on late 40S precursor ribosomes. Proc Natl Acad Sci U S A.

[51] Pober BR (2010). Williams-Beuren syndrome. N Engl J Med.

[52] Petrossian TC, Clarke SG (2011). Uncovering the human methyltransferasome. Mol Cell Proteomics.

[53] Ohbayashi I, Konishi M, Ebine K, Sugiyama M (2011). Genetic identification of Arabidopsis RID2 as an essential factor involved in pre-rRNA processing. Plant J.

[54] Jangani M, Poolman TM, Matthews L, Yang N, Farrow SN, Berry A (2014). The methyltransferase WBSCR22/Merm1 enhances glucocorticoid receptor function and is regulated in lung inflammation and cancer. J Biol Chem.

[55] Yan D, Tu L, Yuan H, Fang J, Cheng L, Zheng X (2017). WBSCR22 confers oxaliplatin resistance in human colorectal cancer. Sci Rep.

[56] Khan AA, Huang H, Zhao Y, Li H, Pan R, Wang S et al. WBSCR22 and TRMT112 synergistically suppress cell proliferation, invasion and tumorigenesis in pancreatic cancer via transcriptional regulation of ISG15. Int J Oncol. 2022;60(3). 10.3892/ijo.2022.5314 PMC8857931 35088887

[57] Chi Y, Liang Z, Guo Y, Chen D, Lu L, Lin J et al. WBSCR22 confers cell survival and predicts poor prognosis in glioma. Brain Res Bull. 2020;161. 10.1016/j.brainresbull.2020.04.024 32380188

[58] Leetsi L, Õunap K, Abroi A, Kurg R. The Common Partner of several Methyltransferases TRMT112 regulates the expression of N6AMT1 isoforms in mammalian cells. Biomolecules. 2019;9(9). 10.3390/biom9090422 PMC6769652 31466382

[59] Galloway A, Cowling VH (2019). mRNA cap regulation in mammalian cell function and fate. Biochim Biophys Acta Gene Regul Mech.

[60] Varshney D, Petit A-P, Bueren-Calabuig JA, Jansen C, Fletcher DA, Peggie M (2016). Molecular basis of RNA guanine-7 methyltransferase (RNMT) activation by RAM. Nucleic Acids Res.

[61] Galloway A, Kaskar A, Ditsova D, Atrih A, Yoshikawa H, Gomez-Moreira C (2021). Upregulation of RNA cap methyltransferase RNMT drives ribosome biogenesis during T cell activation. Nucleic Acids Res.

[62] Gonatopoulos-Pournatzis T, Dunn S, Bounds R, Cowling VH (2011). RAM/Fam103a1 is required for mRNA cap methylation. Mol Cell.

[63] Sonenberg N, Hinnebusch AG (2009). Regulation of translation initiation in eukaryotes: mechanisms and biological targets. Cell.

[64] Chidambaranathan-Reghupaty S, Fisher PB, Sarkar D. Hepatocellular carcinoma (HCC): epidemiology, etiology and molecular classification. Adv Cancer Res. 2021;149. 10.1016/bs.acr.2020.10.001 PMC8796122 33579421

[65] Sung H, Ferlay J, Siegel RL, Laversanne M, Soerjomataram I, Jemal A (2021). Global Cancer Statistics 2020: GLOBOCAN estimates of incidence and Mortality Worldwide for 36 cancers in 185 countries. Cancer J Clin.

[66] Nathan H, Aloia TA, Vauthey J-N, Abdalla EK, Zhu AX, Schulick RD (2009). A proposed staging system for intrahepatic cholangiocarcinoma. Ann Surg Oncol.

[67] Popat K, McQueen K, Feeley TW (2013). The global burden of cancer. Best Pract Res Clin Anaesthesiol.

[68] El-Diwany R, Pawlik TM, Ejaz A (2019). Intrahepatic Cholangiocarcinoma. Surg Oncol Clin N Am.

[69] Stefanska B, Cheishvili D, Suderman M, Arakelian A, Huang J, Hallett M (2014). Genome-wide study of hypomethylated and induced genes in patients with liver cancer unravels novel anticancer targets. Clin Cancer Res.

[70] Tian Q-H, Zhang M-F, Zeng J-S, Luo R-G, Wen Y, Chen J (2019). METTL1 overexpression is correlated with poor prognosis and promotes hepatocellular carcinoma via PTEN. J Mol Med (Berl).

[71] Dai Z, Liu H, Liao J, Huang C, Ren X, Zhu W et al. N-Methylguanosine tRNA modification enhances oncogenic mRNA translation and promotes intrahepatic cholangiocarcinoma progression. Mol Cell. 2021;81(16). 10.1016/j.molcel.2021.07.003 34352206

[72] Parfenov M, Pedamallu CS, Gehlenborg N, Freeman SS, Danilova L, Bristow CA (2014). Characterization of HPV and host genome interactions in primary head and neck cancers. Proc Natl Acad Sci U S A.

[73] Kolbe AR, Bendall ML, Pearson AT, Paul D, Nixon DF, Pérez-Losada M et al. Human endogenous Retrovirus expression is Associated with Head and Neck Cancer and Differential Survival. Viruses. 2020;12(9). 10.3390/v12090956 PMC7552064 32872377

[74] Chen J, Li K, Chen J, Wang X, Ling R, Cheng M (2022). Aberrant translation regulated by METTL1/WDR4-mediated tRNA N7‐methylguanosine modification drives head and neck squamous cell carcinoma progression. Cancer Commun.

[75] Ke L, Xiang Y, Guo X, Lu J, Xia W, Yu Y (2016). c-Src activation promotes nasopharyngeal carcinoma metastasis by inducing the epithelial-mesenchymal transition via PI3K/Akt signaling pathway: a new and promising target for NPC. Oncotarget.

[76] Chen B, Jiang W, Huang Y, Zhang J, Yu P, Wu L (2022). N7-methylguanosine tRNA modification promotes tumorigenesis and chemoresistance through WNT/β-catenin pathway in nasopharyngeal carcinoma. Oncogene.

[77] Antoni S, Ferlay J, Soerjomataram I, Znaor A, Jemal A, Bray F. Bladder Cancer incidence and mortality: A global overview and recent Trends. Eur Urol. 2017;71(1). 10.1016/j.eururo.2016.06.010 27370177

[78] Bamias A, Tzannis K, Harshman LC, Crabb SJ, Wong YN, Kumar Pal S (2018). Impact of contemporary patterns of chemotherapy utilization on survival in patients with advanced cancer of the urinary tract: a Retrospective International Study of Invasive/Advanced Cancer of the urothelium (RISC). Ann Oncol.

[79] Ying X, Liu B, Yuan Z, Huang Y, Chen C, Jiang X et al. METTL1-m 7 G‐EGFR/EFEMP1 axis promotes the bladder cancer development. Clin Translational Med. 2021;11(12). 10.1002/ctm2.675 PMC8694502 34936728

[80] Xie H, Wang M, Yu H, Wang H, Ding L, Wang R (2022). METTL1 drives tumor progression of bladder cancer via degrading ATF3 mRNA in an m7G-modified mir-760-dependent manner. Cell Death Discov.

[81] Torre LA, Bray F, Siegel RL, Ferlay J, Lortet-Tieulent J, Jemal A. Global cancer statistics, 2012. Cancer J Clin. 2015;65(2). 10.3322/caac.21262 25651787

[82] Siegel RL, Miller KD, Jemal A, Cancer statistics. 2015. CA: a Cancer Journal For Clinicians. 2015;65(1). 10.3322/caac.21254 25559415

[83] Wang C, Wang W, Han X, Du L, Li A, Huang G (2021). Methyltransferase-like 1 regulates lung adenocarcinoma A549 cell proliferation and autophagy via the AKT/mTORC1 signaling pathway. Oncol Lett.

[84] Kelsen D (2001). Preoperative chemoradiotherapy for esophageal cancer. J Clin Oncol.

[85] Triantafyllou T, Wijnhoven BPL (2020). Current status of esophageal cancer treatment. Chin J Cancer Res.

[86] Lagergren J (2015). Oesophageal cancer in 2014: advances in curatively intended treatment. Nat Rev Gastroenterol Hepatol.

[87] Han H, Yang C, Ma J, Zhang S, Zheng S, Ling R et al. N7-methylguanosine tRNA modification promotes esophageal squamous cell carcinoma tumorigenesis via the RPTOR/ULK1/autophagy axis. Nat Commun. 2022;13(1). 10.1038/s41467-022-29125-7 PMC8933395 35304469

[88] Han H, Zheng S, Lin S (2023). N7-methylguanosine (m7G) tRNA modification: a novel autophagy modulator in cancer. Autophagy.

[89] Marshall GM, Carter DR, Cheung BB, Liu T, Mateos MK, Meyerowitz JG (2014). The prenatal origins of cancer. Nat Rev Cancer.

[90] Bosse KR, Maris JM (2016). Advances in the translational genomics of neuroblastoma: from improving risk stratification and revealing novel biology to identifying actionable genomic alterations. Cancer.

[91] Huang Y, Ma J, Yang C, Wei P, Yang M, Han H (2022). METTL1 promotes neuroblastoma development through m7G tRNA modification and selective oncogenic gene translation. Biomark Res.

[92] Louis DN, Ohgaki H, Wiestler OD, Cavenee WK, Burger PC, Jouvet A et al. The 2007 WHO classification of tumours of the central nervous system. Acta Neuropathol. 2007;114(2). 10.1007/s00401-007-0243-4 PMC1929165 17618441

[93] Li L, Yang Y, Wang Z, Xu C, Huang J, Li G (2021). Prognostic role of METTL1 in glioma. Cancer Cell Int.

[94] Siegel RL, Miller KD, Jemal A, Cancer statistics. 2016. CA: a Cancer Journal For Clinicians. 2016;66(1). 10.3322/caac.21332 26742998

[95] Tsai M-J, Chang W-A, Huang M-S, Kuo P-L (2014). Tumor microenvironment: a new treatment target for cancer. ISRN Biochem.

[96] Tung KH, Ernstoff MS, Allen C, Shu SL (2019). A review of Exosomes and their role in the Tumor Microenvironment and host-tumor macroenvironment. J Immunol Sci.

[97] Gener Lahav T, Adler O, Zait Y, Shani O, Amer M, Doron H (2019). Melanoma-derived extracellular vesicles instigate proinflammatory signaling in the metastatic microenvironment. Int J Cancer.

[98] Xu F, Cai D, Liu S, He K, Chen J, Qu L (2023). N7-methylguanosine regulatory genes well represented by METTL1 define vastly different prognostic, immune and therapy landscapes in adrenocortical carcinoma. Am J cancer Res.

[99] Chen J, Li K, Chen J, Wang X, Ling R, Cheng M (2022). Aberrant translation regulated by METTL1/WDR4-mediated tRNA N7-methylguanosine modification drives head and neck squamous cell carcinoma progression. Cancer Commun (Lond).

[100] Wang Y-T, Chen J, Chang C-W, Jen J, Huang T-Y, Chen C-M (2017). Ubiquitination of tumor suppressor PML regulates prometastatic and immunosuppressive tumor microenvironment. J Clin Invest.

[101] Italiani P, Boraschi D (2014). From monocytes to M1/M2 macrophages: phenotypical vs. functional differentiation. Front Immunol.

[102] Vignali DAA, Collison LW, Workman CJ (2008). How regulatory T cells work. Nat Rev Immunol.

[103] Kim HR, Park HJ, Son J, Lee JG, Chung KY, Cho NH (2019). Tumor microenvironment dictates regulatory T cell phenotype: upregulated immune checkpoints reinforce suppressive function. J Immunother Cancer.

[104] Ma S, Zhu J, Wang M, Zhu J, Wang W, Xiong Y (2022). Comprehensive analysis of m7G modification patterns based on potential m7G regulators and tumor microenvironment infiltration characterization in lung adenocarcinoma. Front Genet.

[105] Rong J, Wang H, Yao Y, Wu Z, Chen L, Jin C (2022). Identification of m7G-associated lncRNA prognostic signature for predicting the immune status in cutaneous melanoma. Aging.

[106] Liu Y, Yang C, Zhao Y, Chi Q, Wang Z, Sun B (2019). Overexpressed methyltransferase-like 1 (METTL1) increased chemosensitivity of colon cancer cells to cisplatin by regulating miR-149-3p/S100A4/p53 axis. Aging.

[107] Huang M, Long J, Yao Z, Zhao Y, Zhao Y, Liao J et al. METTL1-Mediated m7G tRNA modification promotes Lenvatinib Resistance in Hepatocellular Carcinoma. Cancer Res. 2023;83(1). 10.1158/0008-5472.CAN-22-0963 36102722

[108] Okamoto M, Fujiwara M, Hori M, Okada K, Yazama F, Konishi H (2014). tRNA modifying enzymes, NSUN2 and METTL1, determine sensitivity to 5-fluorouracil in HeLa cells. PLoS Genet.

[109] Mauer J, Luo X, Blanjoie A, Jiao X, Grozhik AV, Patil DP (2017). Reversible methylation of m6Am in the 5’ cap controls mRNA stability. Nature.

[110] Zhang Q, Kang Y, Wang S, Gonzalez GM, Li W, Hui H (2021). HIV reprograms host m6Am RNA methylome by viral vpr protein-mediated degradation of PCIF1. Nat Commun.

[111] Huff S, Tiwari SK, Gonzalez GM, Wang Y, Rana TM (2021). m6A-RNA demethylase FTO inhibitors impair Self-Renewal in Glioblastoma Stem cells. ACS Chem Biol.

[112] Boulias K, Toczydłowska-Socha D, Hawley BR, Liberman N, Takashima K, Zaccara S et al. Identification of the m6Am methyltransferase PCIF1 reveals the location and functions of m6Am in the transcriptome. Mol Cell. 2019;75(3). 10.1016/j.molcel.2019.06.006 PMC6703822 31279658

[113] Liao J, Yi Y, Yue X, Wu X, Zhu M, Chen Y (2023). Methyltransferase 1 is required for nonhomologous end-joining repair and renders hepatocellular carcinoma resistant to radiotherapy. Hepatology.

[114] Zhu S, Wu Y, Zhang X, Peng S, Xiao H, Chen S et al. Targeting N7-methylguanosine tRNA modification blocks hepatocellular carcinoma metastasis after insufficient radiofrequency ablation. Mol Therapy: J Am Soc Gene Therapy. 2022. 10.1016/j.ymthe.2022.08.004 PMC10278047 35965412

[115] Song B, Tang Y, Chen K, Wei Z, Rong R, Lu Z (2020). m7GHub: deciphering the location, regulation and pathogenesis of internal mRNA N7-methylguanosine (m7G) sites in human. Bioinformatics.

[116] Chen W, Feng P, Song X, Lv H, Lin H (2019). iRNA-m7G: identifying N7-methylguanosine Sites by Fusing multiple features. Mol Ther Nucleic Acids.

[117] Bi Y, Xiang D, Ge Z, Li F, Jia C, Song J (2020). An interpretable prediction model for identifying N7-Methylguanosine Sites based on XGBoost and SHAP. Mol Ther Nucleic Acids.

[118] Dai C, Feng P, Cui L, Su R, Chen W, Wei L. Iterative feature representation algorithm to improve the predictive performance of N7-methylguanosine sites. Brief Bioinform. 2021;22(4). 10.1093/bib/bbaa278 33169141

[119] Zou H, Yin Z (2021). m7G-DPP: identifying N7-methylguanosine sites based on dinucleotide physicochemical properties of RNA. Biophys Chem.

[120] Liu X, Liu Z, Mao X, Li Q (2020). m7GPredictor: an improved machine learning-based model for predicting internal m7G modifications using sequence properties. Anal Biochem.

[121] Liu H, Zeng X, Ren X, Zhang Y, Huang M, Tan L et al. Targeting tumour-intrinsic N7-methylguanosine tRNA modification inhibits MDSC recruitment and improves anti-PD-1 efficacy. Gut. 2022. 10.1136/gutjnl-2022-327230 36283801

[122] Mair F, Erickson JR, Frutoso M, Konecny AJ, Greene E, Voillet V (2022). Extricating human tumour immune alterations from tissue inflammation. Nature.

